# Zooming in on Transcription Preinitiation

**DOI:** 10.1016/j.jmb.2016.04.003

**Published:** 2016-06-19

**Authors:** Kapil Gupta, Duygu Sari-Ak, Matthias Haffke, Simon Trowitzsch, Imre Berger

**Affiliations:** 1 European Molecular Biology Laboratory, Grenoble Outstation, 71 Avenue des Martyrs, 38042, Grenoble Cedex 9, France; 2 Unit of Virus Host-Cell Interactions, University of Grenoble Alpes-EMBL-CNRS, UMI 3265, 71 Avenue des Martyrs, 38042, Grenoble, Cedex 9, France; 3 Center for Proteomic Chemistry, Structural Biophysics, Novartis Institute for Biomedical Research NIBR, Fabrikstrasse 2, 4056 Basel, Switzerland; 4 Institute of Biochemistry, Biocenter, Goethe-Universität Frankfurt, Max-von-Laue-Str. 9, 60438 Frankfurt/Main Germany; 5 The School of Biochemistry, University of Bristol, Bristol BS8 1TD, UK

**Keywords:** GTF, general transcription factors, RNA pol II, RNA polymerase II, PIC, preinitiation complex, TBP, TATA-box binding protein, ITC, initially transcribing complex, cryo-EM, cryo-electron microscopy, CLMS, crosslinking mass spectrometry, HFD, histone fold domain, SAGA, Spt–Ada–Gcn5–Acetyl transferase, NIP, nuclear import particle, HAT, histone acetyltransferase, Brf2, TFIIB-related factor 2, CTD, C-terminal domain, TAFs, TBP-associated factors, preinitiation complex, general transcription factor TFIID, transcription initiation, RNA polymerase II, multiprotein complex

## Abstract

Class II gene transcription commences with the assembly of the Preinitiation Complex (PIC) from a plethora of proteins and protein assemblies in the nucleus, including the General Transcription Factors (GTFs), RNA polymerase II (RNA pol II), co-activators, co-repressors, and more. TFIID, a megadalton-sized multiprotein complex comprising 20 subunits, is among the first GTFs to bind the core promoter. TFIID assists in nucleating PIC formation, completed by binding of further factors in a highly regulated stepwise fashion. Recent results indicate that TFIID itself is built from distinct preformed submodules, which reside in the nucleus but also in the cytosol of cells. Here, we highlight recent insights in transcription factor assembly and the regulation of transcription preinitiation.

## Highlights

• Architectural models of human and yeast PIC were proposed.
• Mediator core–ITC complex structure reveals novel interactions.
• TFIID submodule residing in the cytoplasm has been discovered.
• Complex assembly emerges as key concept in transcription regulation.

## Graphical Abstract

**Figure:**
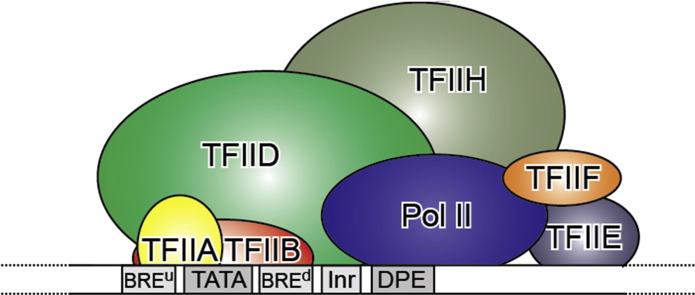

## Introduction

**Fig. 1:**
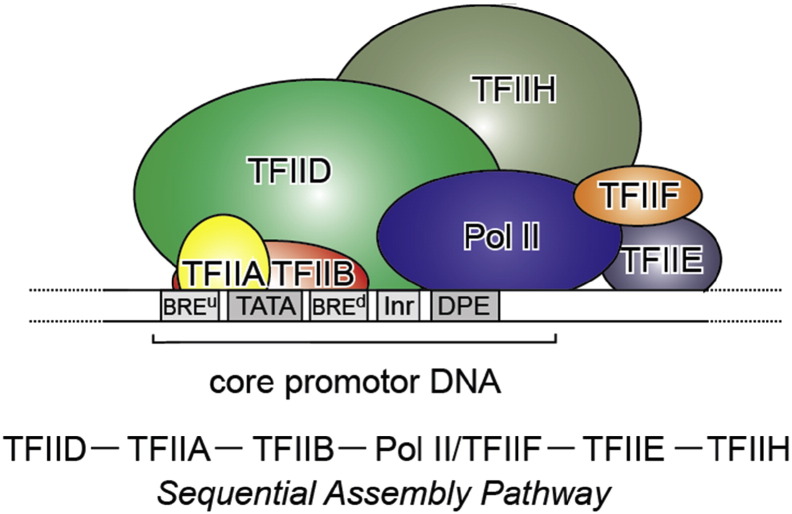
Transcription PIC. Class II gene transcription is brought about by (in humans) over a hundred polypeptides assembling on the core promoter of protein-encoding genes, which then give rise to messenger RNA. A PIC on a core promoter is shown in a schematic representation (adapted from Ref. [5]). PIC contains, in addition to promoter DNA, the GTFs TFIIA, B, D, E, F, and H, and RNA Pol II. PIC assembly is thought to occur in a highly regulated, stepwise fashion (top). TFIID is among the first GTFs to bind the core promoter via its TBP subunit. Nucleosomes at transcription start sites contribute to PIC assembly, mediated by signaling through epigenetic marks on histone tails. The Mediator (not shown) is a further central multiprotein complex identified as a global transcriptional regulator. TATA, TATA-box DNA; BRE^u^, B recognition element upstream; BRE^d^, B recognition element downstream; Inr, Initiator; DPE, Down-stream promoter element.

Class II gene transcription is a tightly regulated, essential process controlled by a highly complex multicomponent machinery. A plethora of proteins, more than a hundred in humans, are organized in often very large multiprotein assemblies including General Transcription Factors (GTFs TFIIA, TFIIB, TFIID, TFIIE, TFIIF, TFIIH), RNA polymerase (RNA pol II), and a large number of diverse complexes that act as co-activators, co-repressors, chromatin modifiers and remodelers (Fig. 1). Class II gene transcription is regulated at various levels: while assembling on chromatin, before and during transcription initiation, throughout elongation and mRNA processing, and termination. A host of activators and repressors has been reported to regulate transcription [1]. A central multisubunit complex called the Mediator was identified as a global regulator of gene expression [2], [3], [4]. Functional and structural analyses of many components of this striking complexity have provided immense insights into the transcription process. In this contribution, we are reviewing, by no means exhaustively, recent important findings about key architectures within the transcription machinery, leading to conceptual advances in terms of complex assembly and function, with a focus on the key GTF that nucleates PIC formation, TFIID.

## PIC Assembly: Lessons from Yeast and Human

Transcription of RNA pol II-dependent genes is triggered by the regulated assembly of the Preinitiation Complex (PIC). PIC formation commences with the binding of TFIID to the core promoter. TFIID contains the TATA-box binding protein (TBP). Binding of TFIID to the core-promoter is followed by the recruitment of further GTFs and RNA pol II. Several lines of evidence suggest that this process occurs in a defined, stepwise order and undergoes significant restructuring [5]. First, PIC adopts an inactive state, the “closed” complex, which is incompetent to initiate transcription. The ATP-dependent translocase activity of the XPB/Ssl2 helicase subunit of GTF TFIIH then opens up about 11 to 15 base pairs around the transcription start site by moving along one DNA strand inducing torsional strain, leading to conformational rearrangements and positioning of single-stranded DNA to the active site of RNA pol II [6], [7], [8], [9]. In this “open” complex, RNA pol II can enter elongation to transcribe throughout a gene in a highly processive manner without dissociating from the DNA template or losing the nascent RNA. In most eukaryotes, after synthesizing about 20–100 bases, RNA pol II can pause (Promoter proximal pause) and then disconnect from promoter elements and other components of the transcription machinery, giving rise to a fully functional elongation complex in a process called promoter escape [10], [11], [12], [13], [14]. The promoter-bound components of the PIC, in contrast, remain in place, and thus only TFIIB, TFIIF, and RNA pol II need to be recruited for re-initiation, significantly increasing the transcription rate in subsequent rounds of transcription [12], [15]. Promoter escape is preceded by an abortive transcription in many systems, where multiple short RNA products of 3 to 10 bases in length are synthesized [16], [17].

Recent landmark studies on human and yeast PIC formation provided more differentiated views of the first steps in the transcription initiation process, corroborating the concept of stepwise assembly while also hinting at significant differences that may be present between the species [18], [19] (reviewed in Ref. [20]). In the study of the human PIC, the proposed assembly mechanism follows the “conventional” stepwise order with the exception that RNA pol II appears to be already recruited at the very beginning, before TFIIF is accreted [18]. According to this model, TFIIF functions in reorganizing the growing PIC, rather than loading RNA pol II into it. TFIIH is the last component to be recruited [18]. According to the model put forward based on the studies from yeast, all GTFs (except TFIIF) including TFIIH assemble into a PIC lacking RNA pol II, which, together with TFIIF, is the last to be incorporated [19]. The structures and models presented in these ground-breaking reports provide a wealth of architectural and functional insight into PIC assembly and convey that there may be different ways to organize PIC in space and time, and that differences between the species may exist [18], [19]. In a separate study, the architecture of a yeast initially transcribing complex (ITC) was determined [21]. ITC is an intermediate complex formed during PIC assembly by RNA pol II, TFIIF, TFIIB, TBP, and DNA, as well as a small nascent RNA [21]. Interestingly, this study revealed similarities with the model of human ITC [18], suggesting that the core architecture of PIC is conserved between yeast and human.

Notably, the described studies above used TBP instead of holo–TFIID. TBP has been shown to suffice for basal transcription, whereas holo–TFIID is required for activated transcription [22], [23]. Therefore, it is conceivable that PIC assembly may follow alternative pathways in activated transcription.

## Mediator Core–RNA Pol II Initially Transcribing Complex

**Fig. 2:**
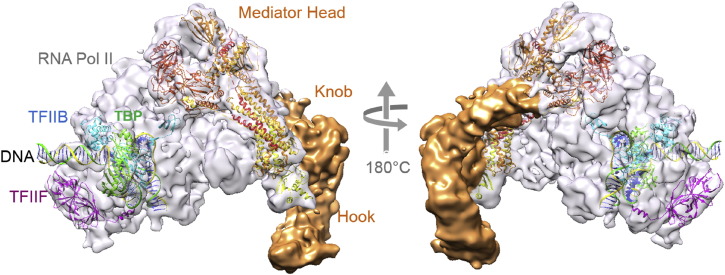
Mediator Core–ITC Architecture. The structure of a Mediator core bound to an initially transcribing RNA pol II-containing complex (EMD-2786; PDB ID: 4V1O) is shown in two views related by a 180° rotation along the vertical axis (arrow). TBP (green), promoter DNA (yellow and green), TFIIB (blue), TFIIF (purple), and the mediator Head module are depicted in a cartoon representation based on X-ray crystal structure coordinates. RNA Pol II is colored in gray. Mediator Middle Module (Knob, Hook) is colored in orange. The structure was determined by cryo-EM combined with CLMS and by fitting of available atomic coordinates (adapted from Ref. [24]).

Recently, single particle cryo-electron microscopy (cryo-EM) and crosslinking mass spectrometry (CLMS) studies of a yeast ITC bound to a Mediator core complex revealed important first insights into transcription initiation and PIC assembly, suggesting that Mediator is involved in stabilizing PIC and in activating RNA pol II [24]. The architecture of Mediator-ITC is shown in Fig. 2. The cryo-EM structure was determined at nanometer resolution, and CLMS proved to be instrumental to decipher the subunit topology of the Mediator Middle module. Mediator interacts with TFIIB and RNA pol II, engaging three distinct interfaces, one of which is thought to be more transient. One interface features an interaction of the so-called mobile jaw of the Mediator Head with the β-ribbon domain of TFIIB and RNA pol II. The second interface is formed by the conserved arm domain of the Head engaging RNA pol II. *In vivo* experiments showed reduced mRNA synthesis when the observed Mediator–TFIIB–RNA pol II interactions were perturbed, confirming the importance of the identified interfaces [20]. These results provide a molecular rationale for the key roles of Mediator in recruiting TFIIB, stabilizing PIC, and activating RNA pol II.

## TFIID Structural Plasticity

**Fig. 3:**
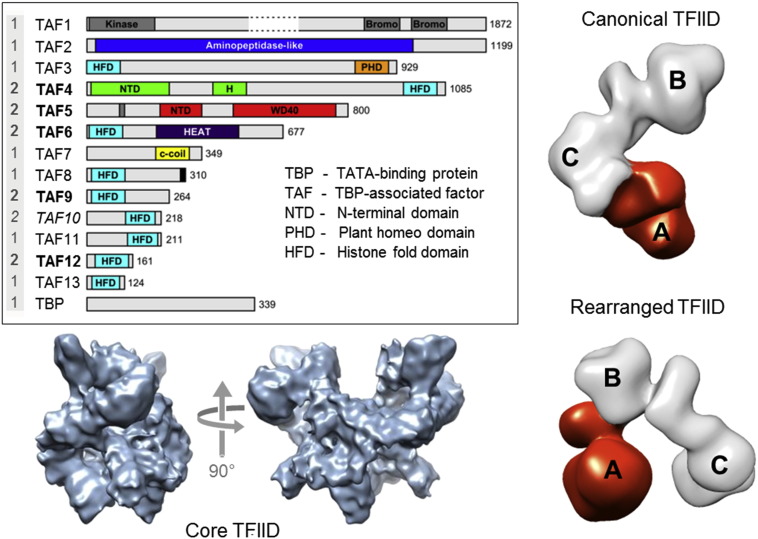
Human GTF TFIID. TFIID is a large megadalton-sized multiprotein complex comprising about 20 subunits made up of 14 different polypeptides. The constituent proteins of TFIID, TBP and the TAFs, are shown in a schematic representation depicted as bars (inset, left). Structured domains are marked and annotated. The presumed stoichiometry of TAFs and TBP in the TFIID holo-complex is given (far left, gray underlaid). TAF10 (in italics) makes histone fold pair separately with both TAF3 and TAF8. TAFs present in a physiological TFIID core complex extracted from eukaryotic nuclei are labeled in bold. The architecture of TFIID core complex (EMD-2230) determined by cryo-EM is shown (bottom left) in two views related by a 90° rotation (arrows) [35]. The holo–TFIID complex is characterized by remarkable structural plasticity. Two conformations, based on cryo-EM data (EMD-2284 and EMD-2287), are shown on the right, a canonical form (top) and a more recently observed rearranged form (bottom). In the rearranged conformation, lobe A (colored in red) migrates from one extreme end of the TFIID complex (attached to lobe C) all the way to the other extremity (attached to lobe B) [33].

A key component in transcription initiation is GTF TFIID, a large megadalton-sized multiprotein complex with around 20 subunits made up of 14 different polypeptides: TBP and the TBP-associated factors (TAFs) (numbered 1–13) (Fig. 3) [25]. Analyses of subunit stoichiometry within TFIID revealed that a number of TAFs are present in two copies while others are found in single copy (Fig. 3) [26]. A key feature in TAFs is the histone fold domain (HFD), which is present in 9 out of 13 TAFs in TFIID. The HFD is a strong protein–protein interaction motif that mediates specific dimerization. The HFD-containing TAFs are organized in discrete heterodimers, with the exception of TAF10, which is capable of forming dimers with two different TFIID components, TAF3 and TAF8. HFDs and several other structural features of TBP and the TAFs are well conserved between the species [27], [28].

TFIID was shown to adopt an asymmetric, horse-shoe shape with three almost equal-sized lobes (A, B, and C), exhibiting a considerable degree of conformational flexibility with at least two distinct conformations (open and closed) [29], [30], [31], [32]. More recent cryo-EM analyses of TFIID in the presence of TFIIA and a synthetic “super core promoter” containing a non-natural combination of promoter elements revealed a novel, completely reorganized conformational state of TFIID (Fig. 3) [33]. In this reorganized state, an entire lobe of TFIID appears to reorganize and migrate to a different position within the holo-complex. A significant number of interactions must be disrupted and reformed in such a large-scale movement, challenging the classical view of TFIID (and similar complexes) as rigidly structured assemblies.

## Nuclear TFIID Core Complex

Further support for the remarkable structural flexibility of TFIID comes from recent studies of a physiological TFIID core complex. Core-TFIID consists of a subset of TAFs, TAF4, TAF5, TAF6, TAF9, and TAF12, and was first identified in *Drosophila* nuclei [34]. Hybrid studies of human core-TFIID integrating cryo-EM, data from X-ray crystallography, and homology models and proteomics revealed a twofold symmetric, intertwined architecture with large solvent channels, formed by two copies each of the constituent TAFs (Fig. 3) [35]. Interestingly, this symmetric structure was shown to reorganize into an asymmetric shape upon binding of a single TAF8–TAF10 heterodimer. The TAF8–TAF10 complex binds in close vicinity to the twofold axis relating the two identical halves of core-TFIID, thereby obstructing the integration of the second copy of TAF8–TAF10 by steric hindrance. These structural rearrangements provide compelling evidence for significant reorganizations in the process of TFIID assembly, which may well channel into large-scale restructuring of the holo-complex when binding to core-promoter DNA and, possibly, other components of the preinitiation machinery.

## Preformed TFIID Submodule in the Cytoplasm

Not much is known to date about the mechanisms of TFIID assembly *in vivo*. Evidence suggests that TFIID formation is a unique and stepwise process, not just a random accretion of different subunits [36]. The classical view of TFIID (and by analogy, also other multiprotein complexes) as a rigidly crafted, “canonical” complex has suffered significant erosion recently. Tissue-specific forms of TFIID have been described, containing distinct isoforms of certain subunits [37]. Partial TFIID complexes containing only subsets of TAFs, such as, for example, nuclear core-TFIID, have been identified *in vivo*, and their unique roles in transcription regulation have been postulated [34], [37], [38], [39], [40], [41], [42]. Data from affinity capture experiments followed by mass-spectrometry indicate that only a fraction of the TFIID material in cells comprises a full complement of TAFs and TBP, while the majority appears to exist in partial complexes containing only a subset of TAFs [43]. Moreover, TAFs are not confined to TFIID—they are also found in the Spt–Ada–Gcn5–Acetyl transferase (SAGA) complex, which is another transcriptional co-activator of similar complexity [44]. Taken together, these findings convey a fluid situation in the nuclei of cells, in which partial TFIID and SAGA complexes may coexist with complete holo–TFIID. However, the organizational and mechanistic details of the underlying dynamics and the possible physiological consequences remain enigmatic.

**Fig. 4:**
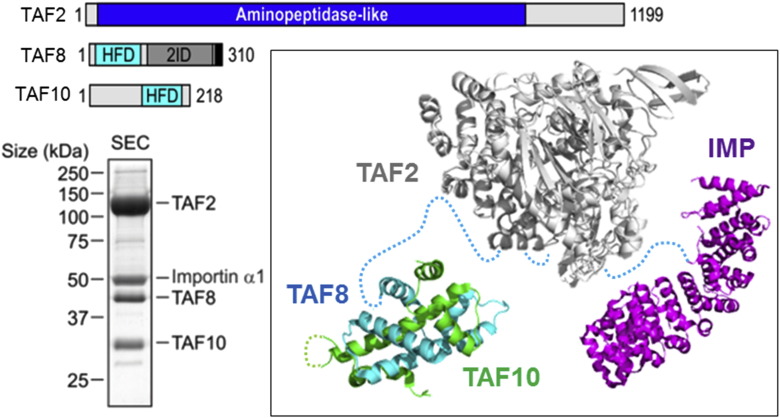
Preformed TFIID Submodule in Cytoplasm. Recently, a stable TFIID subcomplex consisting of TAF2, TAF8, and TAF10 has been discovered, surprisingly residing in the cytoplasm of cells. TAF2, TA8, and TAF10 are shown in a schematic representation (top), depicted as bars. TAF2 is characterized by an extended aminopeptidase-like fold. TAF8 and TAF10 contain HFDs. The TAF8 domain mediating interaction with TAF2 has been identified (marked as 2ID). TAF8 and TAF10 form an HFD pair in the TAF2–8–10 complex. TAF8 contains a nuclear localization signal (black box), and a stable putative nuclear import complex comprising one copy each of TAF2, TAF8, TAF10, and Importin-α could be purified to homogeneity (bottom left). A model of this NIP is shown (inset), based on crystal structures (TAF8–TAF10; TAF8–Imp) (PDB IDs: 4WV4 and 4WV6) and a model of TAF2 threaded on highly homologous human aminopeptides ERAP (adapted from Ref. [45]).

A recent study provided a further piece to this puzzle by reporting the discovery of a novel TFIID submodule formed by TAF2, TAF8, and TAF10 in the cytoplasm of human cells (Fig. 4) [45]. This heterotrimeric complex was dissected by a combination of structural and biochemical methods, *in vitro* and *in vivo* [45]. TAF8 was found to form the backbone of this complex, mediating the interactions with TAF10 by its HFD and with TAF2 by a previously uncharacterized domain in the C-terminal presumably unstructured region of TAF8 (Fig. 4). This TAF2-interaction domain did not only stabilize the TAF2–8–10 complex in the cytosol, but was also shown to be critical for TAF2 accretion into nuclear holo–TFIID, providing new insight into TFIID holo-complex formation. Furthermore, the TAF2–8–10 complex was shown to interact with importin-α via a nuclear localization signal present on TAF8, forming a putative nuclear import particle (NIP) (Fig. 4) [45]. Previous experiments had shown that the import of both TAF8 and TAF10 into the cell nucleus depends on the interaction of TAF8 with importin [46]. Now, it appears that TAF8 not only mediates co-import of TAF10, but also TAF2, by means of the importin α/β pathway and the formation of a TAF2–8–10–Importin NIP.

The existence of stable TFIID subcomplexes in the nucleus (e.g., core–TFIID) and in the cytosol (e.g. TAF2–8–10) supports the concept that preformed TFIID submodules exist, which then combine into the holo-complex, regulated by cellular processes such as nuclear import. It is tempting to speculate that these subassemblies may have holo–TFIID-independent physiological functions, in transcription regulation but possibly also in other unrelated cellular processes. Further study is required to scrutinize and elucidate these exciting possibilities.

## TFIID Domain Structure Gallery

**Fig. 5:**
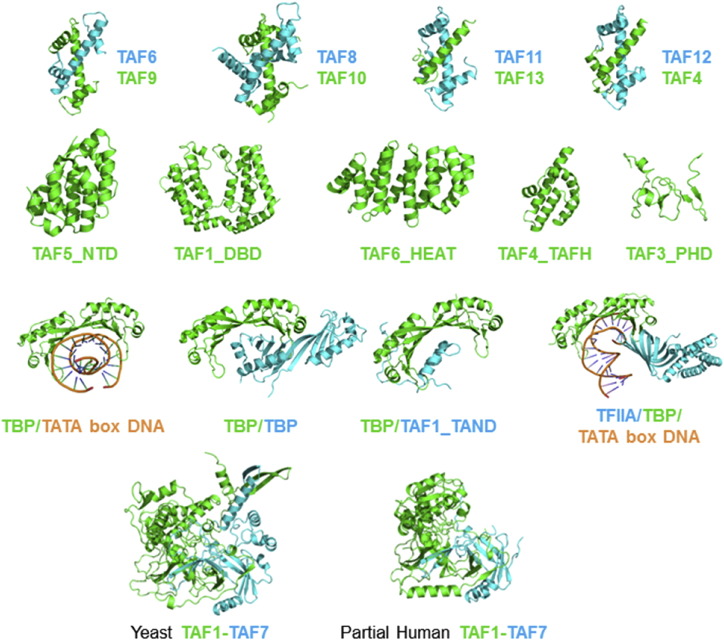
TFIID Domain Structure Gallery. A range of structure of TFIID domains and domain components has been determined at near atomic resolution, mostly by X-ray crystallography. Structures shown here include structures of HFD pairs (top row): *Drosophila* TAF6–TAF9 (PDB ID: 1TAF) [54], human TAF8–TAF10 (PDB ID: 4WV4) [45], human TAF11–TAF13 (PDB ID: 1BH8) [52], and human TAF4–TAF12 (PDB ID: 1H3O) [53]. Crystal structures of isolated single domains of a variety of TAFs have been solved: human TAF5_NTD (PDB ID: 2NXP) [72], human TAF1_DBD (double bromodomain) (PDB ID: 1EQF) [73], the A. locustae TAF6_HEAT repeat (PDB ID: 4ATG) [74], human TAF4_TAFH (PDB ID: 2P6V) [75], and an NMR-based structure of mouse TAF3_PHD (PDB ID: 2K16) [76]. The conserved core of TBP has been studied intensively, and a selection of TBP-containing structures is shown: human TBP in complex with TATA-box DNA (PDB ID: 1CDW) [50], a yeast TBP dimer (PDB ID: 1TBP) [48], a yeast TBP–TAF1_TAND complex (PDB ID: 4B0A) [55], human TBP/TFIIA/DNA complex (PDB ID: 1NVP) [47]. More recently, crystal structures of pairwise interactions within TFIID other than HFDs have been obtained: a yeast TAF1–TAF7 complex (left) (PDB ID: 4OY2) and a partial human TAF1-TAF7 counterpart (right) (PDB ID: 4RGW) were crystallized and their structure determined [51].

Substantial research effort has been dedicated to uncover the molecular structure of TFIID, and much has been learned from the outcomes to date. X-ray crystallography has provided atomic structures of TFIID components, mostly conserved domain(s) in isolation or small complexes with other TAF domains, notably HFDs (Fig. 5). A considerable number of structures of TBP exist, also bound to TFIIA, TFIIB, and TATA-box DNA [47], [48], [49], [50], [51]. Crystal structures of several HFD pairs in TFIID were solved [45], [52], [53], [54]. NMR revealed an interesting case of TATA-box mimicry, with a low-complexity N-terminal domain of TAF1 occupying the DNA binding surface of TBP. This mimicry was later confirmed and expanded by X-ray crystallography [55], [56], [57]. Recently, a crystal structure of a TAF1–TAF7 complex from yeast [58] was determined, followed shortly by a partial structure of human TAF1–TAF7 complex [59]. These structures show that the TAF1–TAF7 interaction is intricate, involving a shared β-barrel motif between TAF1 and TAF7, which both donate β-strands that are intertwined. The β-barrel structure, conserved in yeast and human, is closely reminiscent of the β-barrel found in human TFIIF [60]. Interestingly, a recent study showed that a transcription factor, TFIIIC, which is involved in regulating transcription by RNA pol III, also contains a similar β-barrel fold [61]. These novel findings indicate that this motif may be prevalent in transcription factor complexes and suggest an evolutionary conservation between TFIIF, TFIIIC, and TFIID.

It is noteworthy that the TAF1 fragment used in the TAF1–TAF7 studies encompasses a region that was previously proposed to confer histone acetyltransferase (HAT) activity [62]. However, the corresponding fold in the TAF1–TAF7 crystal structure does not resemble a known HAT domain. It was suggested previously that TAF7 may inhibit the acetyltransferase activity upon binding to TAF1 [56]. Thus, it cannot be ruled out entirely that the binding of TAF7 to TAF1 reconfigures a functional HAT domain into the inactive fold observed in the TAF1–TAF7 model. Evidence suggests that TAF7 may be released from PIC after transcription initiation, when RNA pol II transits to transcript elongation [63], [64]. Phosphorylation of TAF7 by a putative kinase activity contained within TAF1 was proposed to disrupt the TAF1–TAF7 interaction that is thought to result in the TAF7 release from PIC. Specific phosphorylation site(s) in TAF7 were found in its C-terminal part [64], [65], [66]. Interestingly, these are not present in the proposed interaction region between TAF1 and TAF7. Based on the crystal structure, it is unclear how the tight interactions in the TAF1–TAF7 complex would be disrupted by phosphorylation of TAF7, given that the proposed phosphorylation of TAF7 is not within the β-barrel region which would need to be unfolded.

## New Insights from the RNA Pol III System

**Fig. 6:**
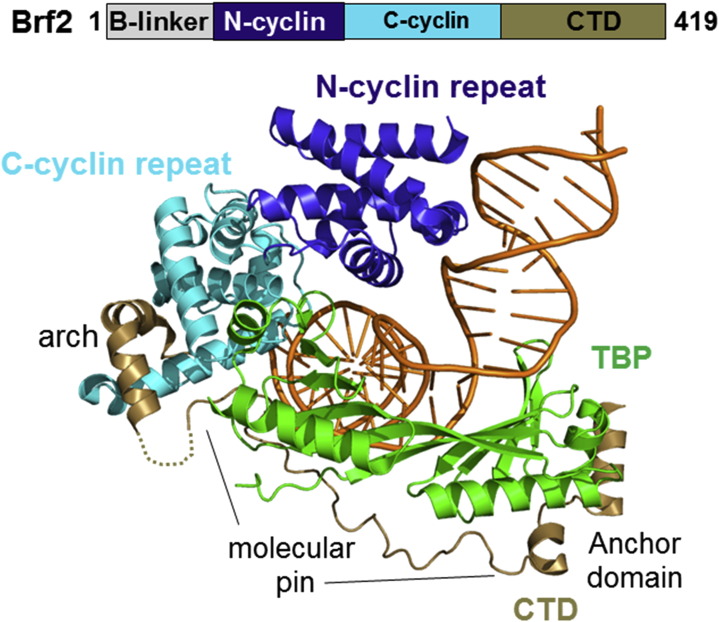
Brf2–TBP–DNA Complex. The crystal structure of Brf2 bound to TBP (green) and promoter DNA (orange) is shown (PDB ID: 4ROC). Brf2 is shown in a schematic representation, depicted as a bar (top). Linker, cyclin domains, and CTD are indicated. The Brf2 CTD reaches over to interact with the convex surface of TBP, mediated by a molecular pin and an anchor domain (adapted from Ref. [67]).

Recently, the structure of TFIIB-related factor 2 (Brf2) in complex with TBP and different natural promoters was determined, providing a detailed view of key interactions in RNA pol III transcription initiation (Fig. 6) [67]. This study validated the proposed structural and functional conservation between TFIIB and TFIIB-related factors [68]. The overall architecture of Brf2–TBP–DNA is similar to the previously determined structures of TFIIB–TBP–DNA [69], [70]. Brf2 is composed of N- and C-terminal cyclin repeats and a C-terminal domain (CTD). The cyclin repeats bind to the minor and major grooves of DNA, respectively, similar to the TFIIB/TBP/DNA ternary structure. The Brf2-specific CTD adopts three distinct structural elements: an “arch”, a “molecular pin” at the Brf2–TBP interface, and a “TBP anchor domain”, which reaches to the convex surface of TBP, reminiscent of TAF1–TBP and Brf1–TBP interactions [55], [71]. Interestingly, in this study, a novel redox-sensing regulatory module was identified in Brf2, involved in redox-dependent control of RNA pol III transcription *in vivo*.

## Conclusions

Unraveling the molecular mechanisms of transcription regulation has fascinated generations of researchers and this trend is unbroken. Advances notably in single particle cryo-EM technology are unlocking molecular structures of multiprotein transcription factors that are forming ever larger and more complex architectures. Crucial new insight about the supramolecular organization of key players, their structural plasticity and unexpected and pronounced dynamic rearrangements are emerging. Classical views of complexes existing as clearly defined, discrete, and uniform entities are challenged by the discovery of multiple isoforms and partial but physiological complexes. Complex assembly from preformed submodules is emerging as a key concept in transcription regulation. We have discussed just a few recent highlights in the present contribution. We anticipate many more thereof in the near future.

## Acknowledgment

We thank all members of the Berger laboratory for helpful discussions. I.B. acknowledges support from the EMBL, the European Commission Framework Programme (FP) 7 ComplexINC project (contract no. 279039), the Agence National de Recherche (ANR) (contract number ANR-13-BSV8-0021) Projet Blanc DiscoverIID, and from the Wellcome Trust (WT106115AIA) through a Senior Investigator Award. M.H. was a Kekulé fellow of the Fonds der Chemischen Industrie (FCI, Germany). S.T. was supported by the European Commission through the Marie-Curie post-doctoral fellowship progamme.
